# Promising New Tools for Targeting p53 Mutant Cancers: Humoral and Cell-Based Immunotherapies

**DOI:** 10.3389/fimmu.2021.707734

**Published:** 2021-08-13

**Authors:** Vitaly Chasov, Mikhail Zaripov, Regina Mirgayazova, Raniya Khadiullina, Ekaterina Zmievskaya, Irina Ganeeva, Aigul Valiullina, Albert Rizvanov, Emil Bulatov

**Affiliations:** ^1^Institute of Fundamental Medicine and Biology, Kazan Federal University, Kazan, Russia; ^2^Institute of Theoretical and Experimental Biophysics of the Russian Academy of Sciences, Pushchino, Russia; ^3^Shemyakin-Ovchinnikov Institute of Bioorganic Chemistry, Russian Academy of Sciences, Moscow, Russia

**Keywords:** p53, mutation, neoantigen, T cell, T cell receptor, T cell receptor mimic antibody, immunotherapy, combined therapy

## Abstract

Transcription factor and oncosuppressor protein p53 is considered as one of the most promising molecular targets that remains a high-hanging fruit in cancer therapy. *TP53* gene encoding the p53 protein is known to be the most frequently mutated gene in human cancers. The loss of transcriptional functions caused by mutations in p53 protein leads to deactivation of intrinsic tumor suppressive responses associated with wild-type (WT) p53 and acquisition of new pro-oncogenic properties such as enhanced cell proliferation, metastasis and chemoresistance. Hotspot mutations of p53 are often immunogenic and elicit intratumoral T cell responses to mutant p53 neoantigens, thus suggesting this protein as an attractive candidate for targeted anti-cancer immunotherapies. In this review we discuss the possible use of p53 antigens as molecular targets in immunotherapy, including the application of T cell receptor mimic (TCRm) monoclonal antibodies (mAbs) as a novel powerful approach.

## Introduction

The tumor suppressor p53 is a protein that performs its cellular functions through transcriptional regulation of genes involved in DNA repair, senescence and apoptosis. The p53 protein is widely known as the “guardian of the genome” that prevents the propagation of cells harboring genetic aberrations, e.g. mutations. *TP53* gene encoding p53 protein is arguably the most frequently altered gene in human cancer (1). The loss of wild-type (WT) p53 functions is the primary outcome of *TP53* mutations that deprives cells of intrinsic tumor suppressive responses, such as senescence and apoptosis. The intracellular p53 level is tightly regulated by its negative regulator murine double minute 2 (MDM2) ubiquitin ligase, primarily through ubiquitination followed by proteasomal degradation. In most human cancers p53 is deactivated either due to loss-of-function mutations or because of the overexpression of MDM2.

The p53 protein is known to trigger immune-related cellular mechanisms and evidence from studying the humoral immune responses in cancer patients testifies that both WT and mutant p53 neoepitopes are recognized by the immune system (2). Recent data revealed that p53 hotspot mutations are immunogenic and elicit intratumoral T cell responses to a range of neoantigens, thus suggesting this protein as an attractive target for anticancer immunotherapies (3).

Antibody-based therapy targets tumor-specific and tumor-associated antigens (TAAs) expressed on the cell surface. However, the majority of such TAAs are localized within the cell which makes them not amenable for such therapies. Intracellular proteins are proteolytically processed by the proteasome to yield 8 to 11 amino acid-long fragments in the cytosol. These peptides are bound in the groove of major histocompatibility complex (MHC) class I molecules, also called human leukocyte antigen (HLA), and presented on the cell surface as peptide/HLA complexes, which enables their recognition by T cell receptors (TCRs) of the T cells. However, the use of soluble TCR domains as therapeutic agents has been hindered by their inherent low affinity and instability as recombinant molecules (4, 5). To this end, T cell receptor mimic (TCRm) antibodies (Abs) recognizing epitopes similar to peptide/HLA complexes have been developed (6–8).

In this review, we discuss the role of p53 (both WT and mutant) in modulation of the immune response during tumor development and its recruitment as a target antigen in immunotherapy, including the novel promising approaches based on TCRm Abs.

## Response of p53 to Immune Signaling

The discovery of p53 in 1979 in association with simian virus 40 (SV40) large T antigen uncovered the crucial role of the protein in viral etiology and immunology of cancer. The joint efforts of the scientific community revealed p53 as the multifaceted molecular actor and resulted in an avalanche of published articles with over 12 000 entries in Pubmed (9).

The p53 protein is an essential component of the innate immune response mediating clearance of damaged cells and defense against external influence (10). The mechanisms of p53 activity involve regulation of the immune landscape by modulating inflammation, senescence and immunity in the surrounding tumor microenvironment (TME), including tumor stroma, extracellular matrix (ECM) and associated immune cells infiltrate (11).

Some immune-associated cellular mechanisms triggered by p53 become dysfunctional when the protein is mutated, and can result in enhanced neoangiogenesis and ECM remodeling, disruption of innate tumor immunity, genotoxic stress response of the toll-like receptor (TLR) pathway, formation of pro-tumor macrophage signature and altered cell-mediated immunity in cancer (12).

Dysfunction of p53 is also associated with the development of autoimmune diseases and often involves overexpression of the *Foxp3* gene in Treg cells (regulatory subpopulation of T cells). TCR signaling was reported to induce upregulation of p53 and downstream transcription activation of *Foxp3* which contributed to p53-mediated Treg cell induction in mice (13).

Cooperation of signals regulating with expression of p53 and induction of natural killer group 2 member D (NKG2D) ligand in tumor cells was associated with their predisposition for immune evasion (14). Additionally, p53 regulates the expression of NKG2D ligands ULBP1 and ULBP2, either positively as a transcriptional target or negatively through the upregulation of miR-34a (11). An important immune checkpoint molecule attenuating the immune response programmed cell death ligand 1 (PD-L1 or CD274) was also found to be regulated by p53. Specifically, p53 modulates the tumor immune response by regulating the expression of miR34, which directly binds to the 3′ untranslated region of the PD-L1 encoding gene (15).

The p53 was also shown to regulate toll-like receptor (TLR) innate immunity genes altering the immune system in response to the DNA stress in cancer cells (16). The human TLR family consists of ten members that regulate adaptor proteins, kinases and effector transcription factors that ultimately induce expression of pro-inflammatory mediators such as cytokines, chemokines and interferons. Targeting of TLR3 and TLR9 by p53 activates their expression and initiates apoptosis (17).

**Figure 1:**
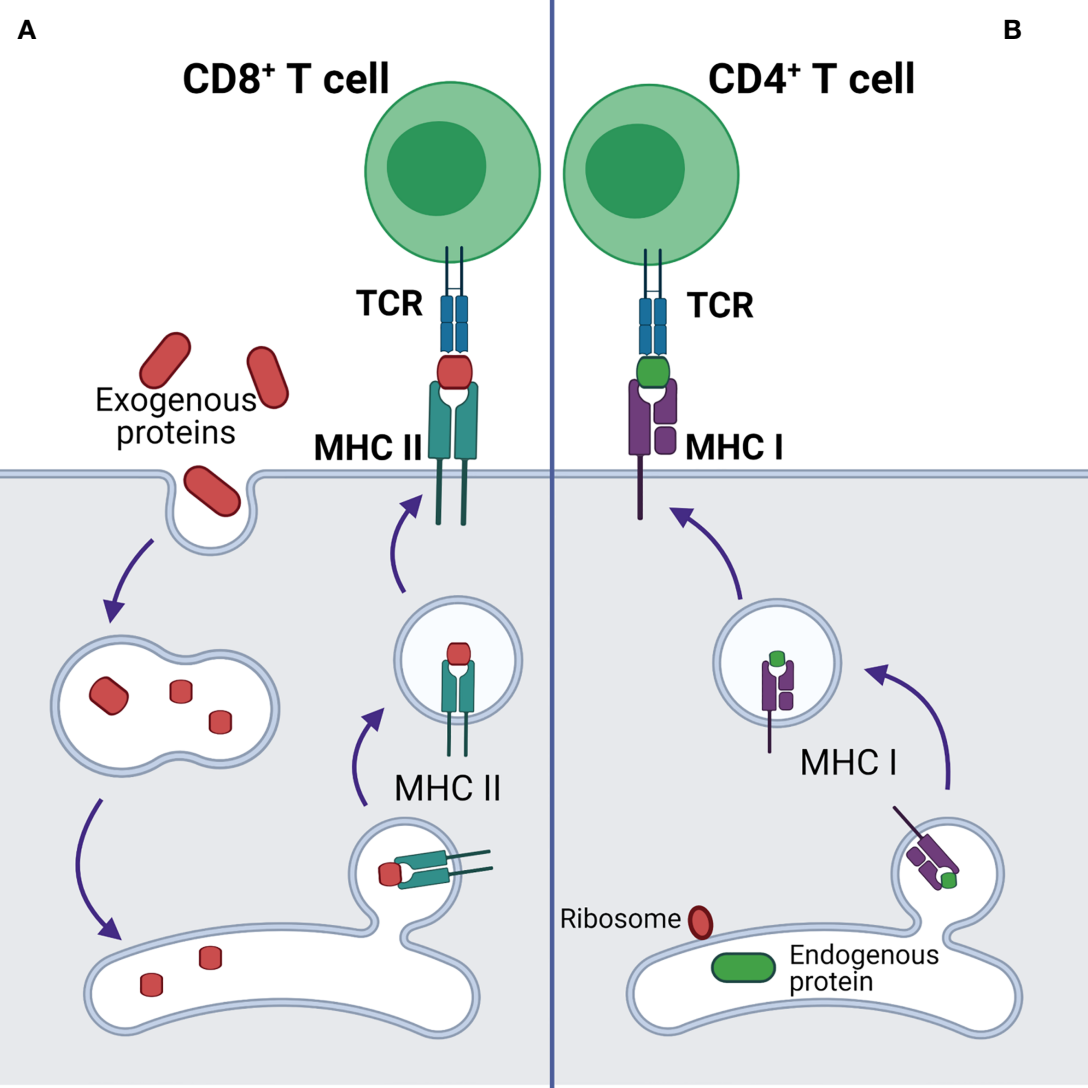
Antigen presentation by MHCI and MHCII complexes. **(A)** Presentation of exogenous antigen to CD4+ T cell by MHCII after lysosomal protein processing. **(B)** Presentation of endogenous antigen (endogenous mutant protein or exogenous protein, e.g. viral protein) to CD8+ T cell by MHCI.

**Figure 2:**
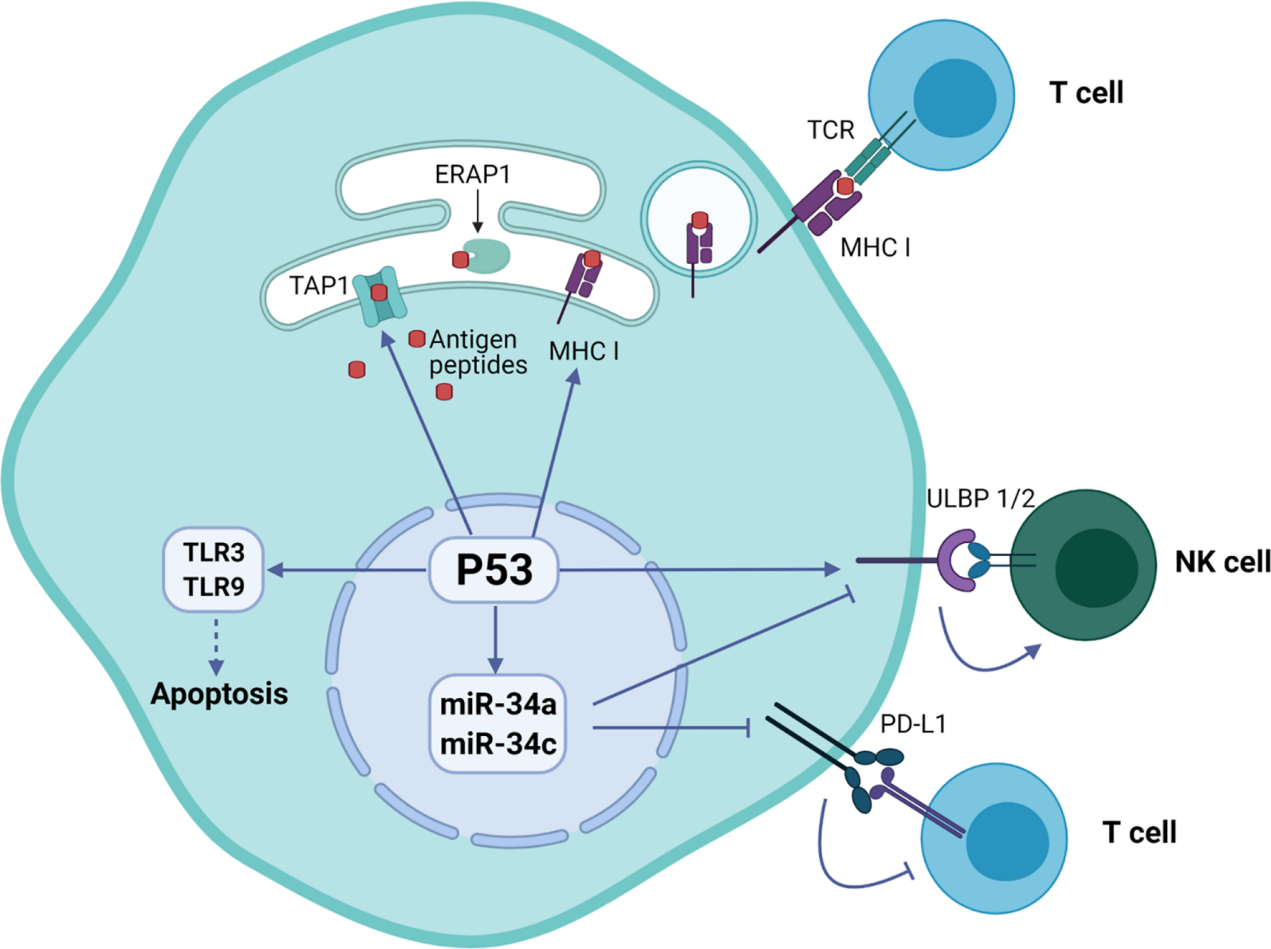
Regulation of immune system functions by p53 protein in tumor cells. The p53 protein is involved in the presentation of endogenous peptides through regulation of TAP1 and ERAP1. In addition, p53 regulates the expression of NKG2D ligands ULBP1 and ULBP2, as well as inhibition of expression PD-L1 ligand through miR-34 microRNA precursor family.

Additionally, p53 regulates endogenous antigen presentation through transcriptional control of aminopeptidase ERAP1 and peptide transporter TAP1. Antigen presentation by MHC class I and class II proteins plays a pivotal role in the adaptive branch of the immune system. Both MHC classes share the task of presenting neoantigen peptides on the cell surface for recognition by T cells. Prior to presentation, peptides are processed from cell’s own endogenous proteins or from exogenous proteins uptaken into the endo-lysosomal system (**Figure 1**). MHCI-associated peptides are generated by proteasomal proteolysis and their translocation into the endoplasmic reticulum requires both TAP1 and TAP2. The p53-driven activation of TAP1 in response to DNA damage increases the pMHCI levels on tumor cells (18). Whereas ERAP1 detaches oligopeptides from the proteasome to ensure their correct length (usually 8-10 amino acids) for MHCI loading (**Figure 2**) (19).

## Adoptive T Cell-Based Immunotherapy

Human cancer is often accompanied by genetic mutations, especially in *TP53*, with each patient carrying their own set of mutations resulting in neoantigen-specific T cell responses. This knowledge can be utilized to develop personalized therapies depending on the tumor genetic profile (20). One of the main treatment modalities within cancer immunotherapy is the adoptive cell therapy (ACT) approach based on autologous or allogeneic tumor-specific cytotoxic T cells. Within the paradigm of this therapeutic approach the cell product is infused into cancer patients with the goal of locating, recognizing and destroying tumor cells (21). Tumor-infiltrating lymphocytes (TILs) represent the oldest branch of ACT, the so-called “blind” approach that includes cultivation, expansion and subsequent transfusion of TILs without their prior selection. Initially TILs are isolated from homogenized tumor tissues or sentinel lymph nodes, then cultured with IL-2 in the presence of tumor lysate as an antigen source and gamma irradiated peripheral blood mononuclear cells (PBMCs) as feeder cells (22). Finally, following the rapid expansion phase (REP) TILs suspension could be infused back into the patient as an autologous cell therapy (23). Adoptive immunotherapy also involves the use of tumor vaccines made from autologous or allogeneic antigen-presenting cells (e.g. dendritic cells) containing private neoepitopes of tumor-associated antigens (24). One of the most prominent and promising examples of ACT is the chimeric antigen receptor (CAR) T cell immunotherapy for the treatment of hematologic B cell malignancies (25, 26).

Neoplastic tumor growth resulting from accumulation of genomic alterations is controlled by the immune system. The mutations often result in translation of abnormal proteins that may be further processed into new immunogenic T cell epitopes (i.e. neoantigens) and serve as potential targets for the T cell based therapies. Neoantigens are short peptides presented on the surface of tumor cells by the pMHC complex. Patient’s own peripheral T cells or TILs may be used as a cell source for the antigen-specific expansion or could be transduced with the artificial TCR specific to the neoantigen of choice. HLA encoding genes are highly variable between individuals and were suggested to a primary role in determining the cancer susceptibility (27). Recent data suggested that the HLA affinity to neoantigen peptides may differ significantly depending on the mutation status unrelated to genotype variation and couldn’t be directly correlated with the immunogenic properties of those neoantigens (28). The issue of neoantigen prediction, identification, and characterization based on genome sequencing data remains unresolved and requires significant efforts at technical and bioinformatic levels.

## Mutant p53 as an Antigen in Cancer Immunotherapy

The *TP53* gene, encoding the p53 tumor suppressor protein, is the most commonly mutated gene in human cancer. Involvement of mutant p53 in malignant inflammation associated with immune dysfunction and the ability of adaptive immune system to respond to mutations in p53 makes this protein an appropriate target for cancer immunotherapy (29). *TP53* missense mutations in pancreatic ductal adenocarcinoma (PDAC) cells were found to increase the extent of fibrosis and reduce the infiltration of cytotoxic CD8+ T cells (30). The inhibition of mutant p53 functions may potentially sensitize PDAC tumors to anticancer treatments, including immunotherapy, therefore reduced infiltration of CD8+ T cells may augment the ability of PDAC tumors to evade the immune system.

Recent data suggest that mutant p53 peptides serve as suitable neoantigens for both CD4+ and CD8+ TCRs (3). The authors employed a high-throughput approach to generate a tandem minigene (TMG) library containing *TP53* mutations that was used to electroporate immature dendritic cells for subsequent co-culturing with TILs. This allowed identification of TILs populations reactive to the mutations frequently occurring at certain p53 hotspots (31). Peripheral blood lymphocytes (PBLs) were isolated from lung cancer patients with mutant p53 (R175H, Y220C, R248W) tumors by sorting antigen-experienced CD4+ and CD8+ T cells. The T cells were then stimulated with mutant p53 peptides *in vitro* to validate the recognition and specificity of the immune response. As a result, T cells with mutant p53-specific TCRs were confirmed to recognize naturally processed p53 neoepitopes *in vitro*. The same research group demonstrated specific T cell responses to *TP53* “hotspot” mutation neoantigens (Y220C, G245S) in patients with metastatic ovarian cancer (32).

Two molecular features often distinguish tumors with mutant p53: overexpression of this otherwise tightly regulated protein and neo-epitope mutations (33, 34). Processed mutant p53 proteins get exposed on the surface of malignant cells as pMHC for immunosurveillance by T cells.

According to the recent data the hepatocellular carcinoma patients carrying *TP53* neoantigens were associated with better prognosis, higher CD8+ lymphocyte infiltration and enhanced immune cytolytic activity (35). Therefore *TP53* neoantigens may affect survival prognosis by regulating anti-tumor immunity and may be considered as promising targets for hepatocellular carcinoma immunotherapy.

The relationship between the tumor mutation burden (TMB), including *TP53* mutations, and clinical relevance was analyzed using the expression data of 546 head and neck squamous cell carcinoma (HNSCC) patients from the Cancer Genome Atlas database (36). The immune-related genes prognostic model was created indicating that high TMB was associated with worse prognosis in HNSCC patients. In addition, macrophages, CD8+ and CD4+ T cells appeared to be the most commonly infiltrated subtypes of immune cells in HNSCC.

The mutant p53-derived peptides have been employed as targets in various immunotherapy strategies some of which are currently in clinical trials (**Table 1**), including anti-cancer vaccines and soluble recombinant TCRs. For example, ALT-801, a biologic drug composed of interleukin-2 (IL-2) genetically fused to a soluble humanized TCR specific to a p53-derived antigen, is currently in phase II clinical trials in combination with gemcitabine (bladder cancer) and cisplatin (metastatic melanoma) (37, 38).

**Table 1:** The list of clinical stage therapies targeting p53 mutant cancers.

Target (Diagnosis)	Therapy	National clinical trial number	Number of patients	Transduced cells/vector	Phase
p53-derived peptides in the context of HLA-A2 (Metastatic melanoma)	ALT-801 (IL-2 genetically fused to a humanized soluble TCR), Cisplatin	NCT01029873	25		II
p53-derived peptides in the context of HLA-A2 (Non-muscle invasive bladder cancer)	ALT-801, Gemcitabine	NCT01625260	52		II
(Metastatic Breast Cancer Malignant Melanoma)	DC vaccine	NCT00978913	31	DCs transfected with mRNA encoding Survivin, hTERT and p53	I
(Head and Neck Squamous Cell Carcinoma Lymphoma)	Recombinant human p53 adenovirus (Ad-p53) with anti-PD-1/anti-PD-L1	NCT03544723	40	Ad-p53	II
(Metastatic breast cancer with mutated p53)	Ad-p53-DC тvaccine, 1-methyl-d-tryptophan	NCT01042535	44	Ad-p53 transduced DCs	II
(Lung Cancer)	Ad-p53-DC vaccine, Nivolumab, Ipilimumab	NCT03406715	14	Ad-p53 transduced DCs	II
(Kidney Cancer) (Melanoma)	Anti-p53 TCR PBLs, Ad-p53-DC vaccine, Aldesleukin	NCT00704938	3	Anti-p53 TCR- transduced PBLs Ad-p53 transduced DCs	II
(Melanoma with p53 overexpression)	Anti-p53 TCR	NCT00393029	12	Anti-p53 TCR- transduced PBLs	II
(Fallopian Tube Carcinoma) (Ovarian Carcinoma) (Peritoneal Carcinoma)	p53-MVA (modified vaccinia Ankara), Pembrolizumab	NCT03113487	28		II

## Therapeutic Monoclonal Antibodies

B and T cells are two classes of lymphocytes playing a key role in the adaptive immune response. Antibodies produced by B cells are usually specific to cell surface or soluble antigens and are unable to penetrate intracellular environment. TCRs recognize target neoantigens in the form of a peptide presented on MHCI or MHCII. The peptides presented on MHCI are normally proteolytic fragments of endogenously processed proteins originating from the cells displaying the pMHCI complex, whereas the peptides on pMHCII usually originate from extracellular proteins taken up and processed by the pMHC-displaying cell through a variety of mechanisms (**Figure 1**) (39).

The specificity and versatility of antibodies has positioned them as highly valuable tools for biological research and various medical applications, including diagnostics and therapy (40). Antibodies and TCRs have high affinities for their pMHC targets in nanomolar and micromolar ranges, respectively (41). Therapeutic monoclonal antibody-based therapy is more flexible and versatile than adoptive T cell-based immunotherapy, since antibodies do not need to be individually tailor-made for each patient and therefore are more accessible at a much lower cost. Antibody therapy also allows easier dosage control and adjusted treatment regimens depending on the patient’s response. Multiple antibody-based drugs such as rituximab, bevacizumab, trastuzumab have proven exceptional utility for cancer therapy (42).

About 50% of all human cancers possess p53 mutations most of which are missense and localized in the DNA-binding domain (DBD) of the protein (1). Most of the mutant p53 proteins are unable to bind DNA and transactivate expression of downstream genes such as *MDM2* which in turn regulates the p53 levels through the autoregulatory loop, thereby resulting in increased levels of the mutant p53 protein in tumor cells (43). Elevated p53 levels can trigger an immune response and cause the production of antibodies (Abs) which appears to be an early event in some cancers (44).

Antibodies against p53 protein have been detected in approximately 17% cases of breast cancer in women (45). In total about 30% of individuals with various cancers were estimated to have detectable anti-p53 Abs (46). High levels of anti-p53 Abs have been detected in patients with premalignant and malignant lesions, and this parameter could be used as a biological marker for early cancer diagnostics (47). Additionally, detection of anti-p53 Abs in saliva has also been reported providing an easier and non-invasive prognostics approach (48).

The anti-p53 Abs usually recognize immunodominant epitopes at both termini of p53, although this is not where the missense mutations are normally located (49). Most of these Abs do not recognize the DBD region where missense mutations often occur and therefore are unable to specifically distinguish between WT and mutant forms of the protein.

## Bispecific Antibodies

Bispecific antibodies (BsAbs) represent a class of monoclonal Abs capable of simultaneous binding two antigens. A subtype of BsAbs called bispecific T cell engagers (BiTEs) has been developed to simultaneously bind tumor-expressed antigens (e.g. BCMA, CD19) and CD3 on T cells (50). The BiTE-mediated interaction of tumor cell with cytotoxic T cell activates proliferation of the latter, thereby increasing the overall number of effector T cells and strengthening the lysis of malignant tumor cells. BiTEs were demonstrated to form such cytolytic synapse with CD8 T cells in a manner independent from MHCI expression on tumor cells (51).

The BiTE binding domains are represented by two single-chain variable fragment (scFv) regions of monoclonal antibodies joined by a flexible peptide linker. One scFv binding domain can be modified to target the surface antigen of interest, while the other domain is always specific to CD3 of TCR. Blinatumomab was the first BiTE approved by the US Food and Drug Administration to treat acute lymphoblastic leukemia (52).

Multiple varieties of the BiTE approach were also developed to diversify the landscape of targeted therapies. These include dual affinity retargeting antibodies (DARTs), as well as bi- and tri-specific killer cell engager antibodies (BiKEs and TriKEs) (51). DARTs use a diabody backbone with the addition of a C-terminal disulfide bridge for improved stabilization. When compared to their equivalent BiTEs CD19-specific DARTs yielded a stronger B cell lysis and T cell-activation (53). BiKEs utilize the innate immune system by harnessing natural killer (NK) cells *via* CD16. Similar to BiKEs, TriKEs consist of a bispecific antibody that recognizes CD16 on NK cells and CD33 on myeloid cancer cells, and in addition they also contain a modified human IL-15 crosslinker (54).

## TCR Mimic Antibodies as an Innovative Class of Therapeutics

A novel class of antibodies binding pMHC often referred to as TCR mimic (TCRm) or TCR-like antibodies represent a highly promising therapeutic modality against cancers associated with mutant p53 (55). In contrast to therapeutic Abs that usually bind soluble or cell surface antigens, the TCRm Abs provide a complementary strategy by effectively targeting the pMHC complexes that present the processed target neoantigen peptides. In recent years multiple TCRm Abs have been developed to target various tumor antigen epitopes in the context of MHC (56, 57). In addition, TCRm Abs have also been explored as candidates for delivery of antibody drug conjugates (ADCs) since pMHC-TCRm Ab complexes can be effectively internalized (58).

## TCR Mimic Antibodies in Cancer Immunotherapy

The cell surface abundance of pMHC complexes for efficient presentation of neoantigens is often a topic of debate (8, 59–61). In general, mAbs are widely used to treat a wide range of diseases, whereas TCRm Abs have not yet been approved for the therapeutic use. This might be a consequence of low-throughput generation of new candidates and their insufficient initial quality that requires laborious downstream refinement.

The development and production of high-affinity, antigen-specific TCRm Abs is highly complex and requires substantial efforts for setting up the manufacturing processes. Provided rather limited number of dominant HLA alleles within a particular ethnic group targeting the p53 (mutant or WT) associated pMHC ligandome leads to an assumption that this therapeutic approach could be implemented as a finite set of the «off-the-shelf» products.

One of the key starting points is selection of the correct antigens (immunogens) that is exposed on the cell surface as pMHCI. Therefore, histocompatible cells expressing such antigens can be used both as immunogens in hybridoma technologies (murine, rat, rabbit) and as a source of antigens for screening the antibody producers.

The APCs can be programmed for expression of pMHC using vector-based approaches (62, 63) or modern CRISPR-based genome-editing techniques (64, 65). Off- target toxicity issues may be resolved by testing in humanized animal models or using cell reprogramming tools to generate different types of tissues for using them as antigen-bearing surrogates or organoids (66). Other options include commercial specificity screening platforms such as developed by Retrogenix Ltd (United Kingdom) for receptor identification, target deconvolution and off-target profiling (67).

**Figure 3:**
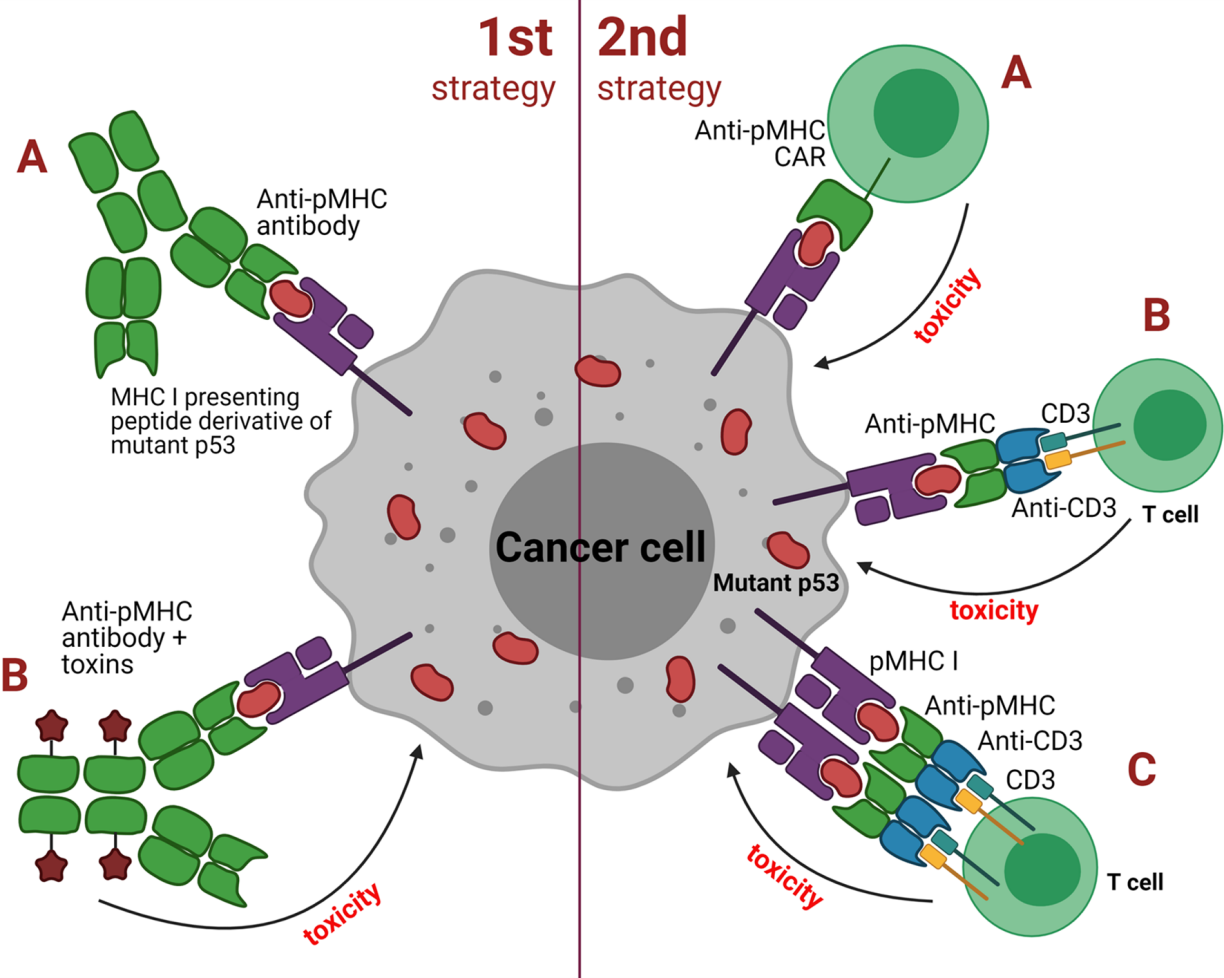
Two strategies employed by TCR mimic antibodies against cancer cells with mutant p53. First strategy: **(A)** classical soluble antibodies for binding to pMHC to induce direct apoptosis or targeted destruction of the tumor cell; **(B)** antibody drug conjugates (ADCs) such as effector molecules, cytokines, toxins or radioactive substances that are coupled to the antibody and upon binding to pMHC result in tumor cell death. Second strategy: **(A)** anti-pMHC CAR to redirect T cells to recognize and lyse tumor cells *via* the scFv fragment derived from a TCR mimic antibody; **(B)** bispecific molecules that bridge cytotoxic T or NK cells with pMHC of the antigen-presenting tumor cell using of the scFv fragment of a TCR mimic antibody; **(C)** similar to B but employs dimeric bispecific T cell-engaging tandem scFv antibodies.

Approaches based on TCRm Abs can be broadly grouped into two major categories depending on the antibody isotype: 1) strategies utilizing classical, soluble antibodies, e.g. for delivering a cytotoxic payload or Fc-mediated recruitment of effector cells or other functional molecules; 2) strategies utilizing redirection of cytotoxic cells (e.g. T or NK cells) or their cooperation with APCs (**Figure 3**). The first category TCRm Abs upon binding to pMHCI initiate assembly of the membrane attack complex (MAC), antibody-dependent cell-mediated cytotoxicity (ADCC) or even trigger the apoptosis. The second category TCRm Abs can be engineered to additionally express CARs that combine intracellular TCR signaling domains and extracellular Fv regions of the antibodies to confer target specificity. CARs are formed by single-chain variable fragments (scFv) capable of redirecting T cells to specifically recognize target antigens and lyse cancer cells. CARs do not directly compete with native TCRs, instead they provide supportive co-stimulation of the cytotoxic signaling cascades. The combination of CAR-T cell therapy with TCR-like antibodies might significantly increase the overall therapeutic potential of this approach.

Alternatively, cytotoxic T cells can be recruited indirectly *via* heterodimeric molecules such as bispecific T cell engagers (BiTEs) that have specificity for pMHC of the target cells and CD3 of T or NK cells. Recent studies reported encouraging data on using this type of immunotherapy against p53-mutant tumors. TCRm Abs specific to pMHC presenting WT and mutant p53 antigens have demonstrated encouraging anti-tumor effects both *in vitro* and *in vivo* in animal models (55, 68).

An interesting example of the BiTE approach is based on bispecific TCRm Ab that recognizes cancer cells expressing the p53(R175H) neoantigen (61). One domain of this antibody recruits TCR and the other binds the pMHC presenting the mutant p53 antigen. In mouse models of multiple myeloma, the BiTEs effectively stimulated T cells to destroy cancer cells bearing mutant p53 without affecting the normal cells with WT p53. Even when the p53 target was presented on the surface of the tumor cells at “extremely low” levels the BiTEs were still able to activate specific T cell-mediated antitumor response. Thus, the employment of TCRm Abs could be potentially useful to target cancers with somatic p53 mutations in addition to other approaches (69).

TCRm Abs were also reported to be designed as bispecific antibodies in single-chain diabody format that demonstrated substantial specificity towards cancer cells expressing neoantigens of the mutant Ras protein (G12V and Q61H/L/R) in mouse models (70). The authors suggested that many TCRm Abs grafted into an optimized BiTE format might be capable of specifically recognizing and destroying cancer cells bearing low levels of the cognate antigens. This could be a highly attractive approach even compared to CAR-T cell therapy that typically requires up to a few thousands of antigen molecules on a single tumor cell for efficient recognition and cytolysis. Worth noting that as opposed to the conceptually preceding TCR approach the TCRm Ab affinity may reach picomolar levels when developed using animal hybridoma technology.

In addition to the above mentioned, CAR-T cell therapy requires a complex and time consuming manufacturing process which significantly limits its broad availability, whereas TCRm Abs if approved are expected to be much more affordable. Another complication of CAR-T cell therapy is the requirement for lymphodepletion prior the infusion (71). As opposed to CAR-T cell therapy, TCRm Ab was not developed to be a personalized treatment. Instead, TCRm Ab therapies link endogenous T cells to tumor-expressed antigens and activate the cytotoxic potential of a patient’s own T cells to eliminate cancer. Also, compared to cell-based immunotherapies antibodies appear much more widely applicable owing to the simplicity of application, reproducibility of results and scalability for mass production. Finally, TCRm Abs can be designed to target both tumor-associated antigens (TAAs) and tumor-specific antigens (TSAs) which fit well with the character of p53 expression in the majority of tumors.

In many cases p53 mutations were associated with significant overexpression of immune checkpoint proteins, such as PD-1, which suggests these types of tumors might be amenable for anti–PD-1/PD-L1 immunotherapy in addition to others approaches (72).

## Conclusion

The p53 protein is an important part of the innate immune and anti-tumor responses. Mutations of p53 often result in loss of its transcriptional activity and therefore inability to regulate anti-tumor and immunomodulatory responses. The peptide neoantigens from a proteolytically processed mutant p53 protein are presented by APCs to B and T cells to activate the immune response. Novel cell-based and humoral immunotherapies will offer previously unavailable levels of medical precision in targeting specific types of tumors. Adoptive T cell-based immunotherapies such as TILs, CAR-T or TCR-T cells may be applied for the treatment of a wide range of tumors. Genome-wide screenings will assist the identification of multiple mutant p53 neoantigens amenable for therapeutic targeting. However, it is important to keep in mind that transgenic TCRs require careful testing for potentially toxic cross-reactivity and might need additional modifications to prevent mispairing with cognate TCRs.

Expanding the target repertoire of therapeutic antibodies to a broad variety of pMHC complexes will offer opportunities for the development of new anticancer strategies and improved treatments. TCR-mimic antibodies can transform the fine cellular specificity of the T cell recognition machinery into a flexible immunotherapeutic approach that fits well in the growing field of personalized medicine. The vast plethora of potential targets represented by a range of mutant p53 neoantigens within the context of the pMHC complexes suggests that TCR-mimic antibodies will find an important place as highly promising immunotherapeutics.

## Author Contributions

VC, MZ, and EB conceived the idea and coordinated the writing. MZ contributed to section about TCR mimic antibodies. RM and RK contributed to section about antibodies. AV and EZ contributed to section about adoptive cell therapy. EZ and IG prepared the figures and table. VC, AR, and EB contributed to introduction and conclusion. All authors contributed to the article and approved the submitted version.

## Funding

Work was funded by RSF grant 19-74-10022 to EB and performed according to the Russian Government Program of Strategic Academic Leadership (Priority 2030) of Kazan Federal University. AV was supported by stipend of the President of Russian Federation CΠ-227.2019.4.

## Conflict of Interest

The authors declare that the research was conducted in the absence of any commercial or financial relationships that could be construed as a potential conflict of interest.

## Publisher’s Note

All claims expressed in this article are solely those of the authors and do not necessarily represent those of their affiliated organizations, or those of the publisher, the editors and the reviewers. Any product that may be evaluated in this article, or claim that may be made by its manufacturer, is not guaranteed or endorsed by the publisher.

## Abbreviations

ACT, adoptive cell therapy; ADC, antibody drug conjugate; ADCC, antibody-dependent cell-​mediated cytotoxicity; APC, antigen-presenting cell; BiKE, bispecific killer cell engager antibody; BiTE, bispecific T cell engager; BsAb, bispecific antibody; CAR, chimeric antigen receptor; CRISPR, clustered regularly interspaced short palindromic repeats; DART, dual affinity retargeting antibody; DBD, DNA binding domain; DC, dendritic cell; ECM, extracellular matrix; ERAP1, endoplasmic reticulum aminopeptidase 1; HC, heavy chain; HLA, human leukocyte antigen Ig, immunoglobulin; IL-2, interleukin 2; mAb, monoclonal antibody MDM2, murine double minute 2; MAC, membrane attack antibody; MHC, major histocompatibility complex; NK, natural killer; NKG2D, natural killer group 2 member D; PBL, peripheral blood lymphocyte; PBMC, peripheral blood mononuclear cell; REP, rapid expansion phase; scFv, single-chain variable fragment; SV40, simian virus 40; TAA, tumor-associated antigen; TCR, T cell receptor; TCRm, T cell receptor mimic; TIL, tumor-infiltrating lymphocyte; TLR, toll-like receptor; TME, tumor microenvironment; TMG, tandem minigene; TriKE, trispecific killer cell engager antibody; TSA, tumor-specific antigen.
